# ER-mitochondria contact sites in neurodegeneration: genetic screening approaches to investigate novel disease mechanisms

**DOI:** 10.1038/s41418-020-00705-8

**Published:** 2020-12-17

**Authors:** Emma Louise Wilson, Emmanouil Metzakopian

**Affiliations:** 1 UK Dementia Research Institute, Department of Clinical Neuroscience, University of Cambridge, Cambridge, CB2 0AH UK; 2 Open Targets, Wellcome Genome Campus, Hinxton, Cambridge, CB10 1SA UK

**Keywords:** Cell biology, Neural ageing, Neurological disorders

## Abstract

Mitochondria-ER contact sites (MERCS) are known to underpin many important cellular homoeostatic functions, including mitochondrial quality control, lipid metabolism, calcium homoeostasis, the unfolded protein response and ER stress. These functions are known to be dysregulated in neurodegenerative diseases, including Parkinson’s disease (PD), Alzheimer’s disease (AD) and amyloid lateral sclerosis (ALS), and the number of disease-related proteins and genes being associated with MERCS is increasing. However, many details regarding MERCS and their role in neurodegenerative diseases remain unknown. In this review, we aim to summarise the current knowledge regarding the structure and function of MERCS, and to update the field on current research in PD, AD and ALS. Furthermore, we will evaluate high-throughput screening techniques, including RNAi vs CRISPR/Cas9, pooled vs arrayed formats and how these could be combined with current techniques to visualise MERCS. We will consider the advantages and disadvantages of each technique and how it can be utilised to uncover novel protein pathways involved in MERCS dysfunction in neurodegenerative diseases.

## Facts

- Mitochondria can form contacts with the ER to regulate vital cellular homoeostatic functions.
- A range of mitochondria-ER contact site (MERCS) tethering proteins maintain these contacts and facilitate their functions. Inositol 1,4,5-triphosphate receptor (IP_3_R), voltage-dependent anion channel (VDAC), glucose-related protein 75 (GRP75) and deglycase (DJ-1) act as a tetramer to regulate Ca^2+^ homoeostasis. Mitofusin-2 (MFN2) and mitofusin-1 (MFN1) can act as a physical tether, control Ca^2+^ homoeostasis and regulate mitochondrial morphology changes. Vesicle-associated membrane protein B (VAPB) and protein tyrosine phosphatase-interacting protein-51 (PTPIP51) are physical tethers and can impact Ca^2+^ homoeostasis, while B-cell receptor-associated protein-31 (BAP-31) can regulate apoptosis.
- MERCS are dysfunctional in neurodegenerative diseases, including Alzheimer’s disease (AD), Parkinson’s disease (PD) and amyloid lateral sclerosis (ALS).
- Pooled or arrayed high-throughput screening can be used in conjunction with CRISPR/Cas9 or RNAi to identify novel disease-relevant pathways in many neurodegenerative diseases.
- The gold standard for visualising MERCS is electron microscopy and super-resolution techniques, but these require vast quantities of sample or image processing and so are not suitable for high-throughput screening.
- A range of split fluorescent protein constructs have been developed, which can be used to visualise MERCS in real time, and are suitable for high-throughput screening.

## Open questions

- What are the molecular mechanisms that govern MERCS dysfunction in neurodegenerative diseases such as AD, PD and ALS? Is there an overarching disease mechanism related to MERCS?
- How can electron microscopy, super resolution, split FP techniques and Ca^2+^ indicators be adapted and optimised for high-throughput screening and which of them will provide the most accurate and efficient readout?

## Introduction

For many years, the textbook view of organelles has been that they function independently and in isolation from each other, forming separate contained entities within a cell. Now, however, contacts between organelles are shown to play fundamental roles in many aspects of cellular health. Membrane contact sites are sections of two adjacent membranes that are in close proximity, but do not fuse. They provide ‘hotspots’ for lipid and ion transfer, as well as signalling and cross talk between organelles. One of the most well-studied membrane contact sites is the mitochondria-ER contact site (MERCS). MERCS was originally identified by electron microscopy (EM) of rat tissue in the 1950s [1], and typically has a diameter of 10–80 nm. The diameter of MERCS can fundamentally affect its functions, which include lipid metabolism, calcium (Ca^2+^) homoeostasis, unfolded protein response (UPR), ER stress and mitochondrial quality control (MQC), all of which have been implicated in neurodegenerative diseases such as Alzheimer’s disease (AD), Parkinson’s disease (PD) and amyotrophic lateral scoliosis (ALS) [2]. However, much work is still needed to elucidate how MERCS affects the progression of neurodegeneration [3].

This review aims to update the field on the molecular composition and functions of MERCS, with specific focus on its impact in AD, PD and ALS. We will then discuss techniques used to study MERCS and evaluate their suitability in high-throughput screening (HTS) to identify novel protein targets involved in MERCS regulation and neurodegenerative diseases.

## The molecular basis of ER-mitochondria contact sites

**Fig. 1:**
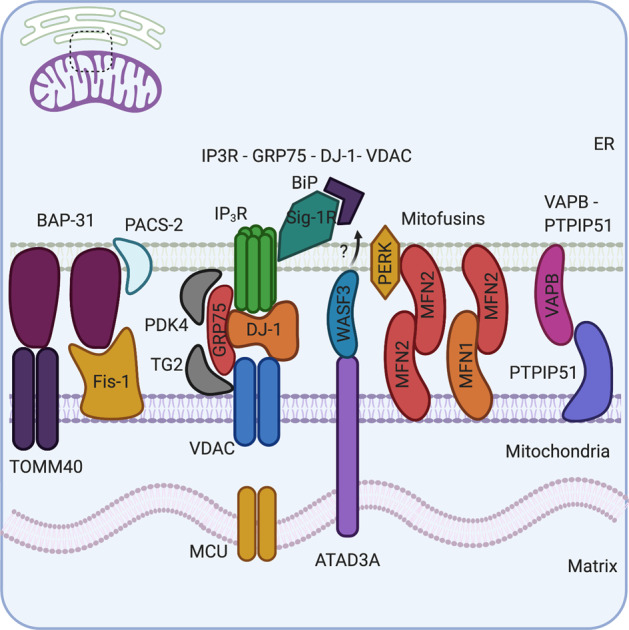
The molecular composition of mitochondria-ER contact sites: several sets of complexes tether the mitochondria and ER. BAP-31 in the ER interacts with Fis-1 and TOMM40 in the OMM. PACS-2, a multifunctional sorting protein in the ER, regulates BAP-31 MERCS interactions. IP_3_R in the ER and VDAC in the OMM form a tetramer complex with regulatory proteins, GRP75 and DJ-1, to control calcium (Ca^2+^) transfer into the mitochondria. Further regulatory proteins such as TG2 and PDK-4 also bind GRP75–IP_3_R–VDAC complex regulating MERCS. Sig-1R accumulates in MERCS and can stabilise IP_3_R in MERCS, but also interacts with chaperone protein BIP in the ER lumen. ATAD3A can cross the IMM and OMM to interact with BiP in the ER via the cytosolic protein WASF3 and other unknown proteins. MFN2 is located in both the ER and mitochondrial membrane and can homodimerise with itself or heterodimerise with MFN1 in the OMM. MFN2 has known interactions with PERK, which are required for progression through the UPR. Finally, VAPB in the ER membrane and PTPIP51 in the OMM interact and directly regulate MERCS size and length and act as a physical tether.

EM shows small black rods reaching between the ER and mitochondrial membrane, these were found to be molecular bridges pinning the two membranes together. These molecular bridges are composed of tethering proteins, and studies have shown a huge variety of molecular tethers, ranging from Ca^2+^ channels to apoptotic proteins [4] (Fig. 1).

### IP3R–DJ-1–GRP75–VDAC

The first tethering complex identified was between the voltage-dependent anion channel (VDAC) (an outer mitochondrial membrane (OMM) protein) and the ER-residing inositol 1,4,5-triphosphate receptor (IP_3_R). This interaction is regulated by glucose-related protein 75 (GRP75) (a molecular chaperone) and deglycase (DJ-1), a protein mutated in PD, that has been revealed to interact with VDAC, IP_3_R and GRP75 [5, 6]. Together, IP_3_R, VDAC, GRP75 and DJ-1 act as a tetramer complex to regulate the transfer of Ca^2+^ from the ER to the mitochondrial matrix via the mitochondrial calcium uniporter (MCU). Interestingly, complete loss of IP_3_R did not show a physical alteration in MERCS; however, the loss of GRP75 or DJ-1 abolishes the Ca^2+^ influx into the mitochondria [5–7] suggesting that the tetramer of VDAC, IP_3_R, GRP75 and DJ-1 acts as a functional tether, rather than a physical one [4].

### Mitofusins

Mitofusin-2 (MFN2) is one of the most well-studied, yet most controversial, MERCS tethers. MFN2 is known for its role in mitochondrial fusion; however, it also localises at ER membranes and can hetero- or homodimerise with mitofusin-1 (MFN1) or MFN2 in the OMM. The Scorrano group first described MFN2 as a physical tether between the ER and mitochondria; however, these results have been called into question owing to the analysis methods used [8]. Other groups have shown that knockdown of MFN2 results in an increase in MERCS, with accompanying increases in Ca^2+^ transfer, supporting the notion that MFN2 is not a physical tether [9, 10]. Most recently, however, the Scorrano group provided new evidence, using split fluorescent protein (FP) systems, to demonstrate that loss of MFN2 causes a decrease in the distance between ER and mitochondria membranes, impairing Ca^2+^ uptake into the mitochondria [11]. Overall, it is accepted that MFN2 is a key component of MERCS and is important in the appropriate functioning of MERCS.

### VAPB and PTPIP51

Vesicle-associated membrane protein B (VAPB) is an ER-residing protein that interacts with protein tyrosine phosphatase-interacting protein-51 (PTPIP51) in the OMM [12]. EM and confocal microscopy show that alterations in PTPIP51 or VAPB are accompanied by changes in the proportion of ER in contact with the mitochondria; thus, loss of these proteins can decrease MERCS [13]. The interactions between VAPB and PTPIP51 directly affect the function of MERCS, as depletion of either can disturb Ca^2+^ handling, resulting in a delay in Ca^2+^ uptake by the mitochondria. Furthermore, a VAPB mutant, which decreases Ca^2+^ handling, also shows aggregation of the mitochondria [12–14]. Together, these data suggest that MERCS impacts on the health of the mitochondria, and this can be regulated by the VAPB–PTPIP51 tether complex

### BAP-31

B-cell receptor-associated protein-31 (BAP-31) is a 28-kDa, ER-residing, membrane chaperone that can physically interact with mitochondrial fission protein-1, serving as a platform to promote recruitment and activation of procaspase 8 and the transmission of pro-apoptotic signals from the mitochondria to the ER. This interaction is also present in non-apoptotic cells suggesting that it forms a preformed scaffold complex [15, 16]. In addition, loss of phosphofurin acidic cluster sorting protein-2 (PACS-2), which can regulate the ER-mitochondria axis, results in BAP-31- mediated fragmentation of the mitochondria, depletion of Ca^2+^ signal and a decrease in MERCS itself [17]. Furthermore, BAP-31 has been shown to regulate mitochondrial oxygen consumption, autophagy and maintain mitochondrial homoeostasis through interactions with TOMM40, mitochondrial respiratory chain complexes and NADH: ubiquinone oxidoreductase (mitochondrial complex 1) core subunit 4 (NDUFS4) located in MERCS [18]. These data suggest that BAP-31 can act as a MERCS tether and a platform for transmittance of apoptotic signals between ER and mitochondria.

### Other MERCS regulatory proteins and tethering components

In addition to the previously discussed protein tethers, MERCS has a wide range of regulatory proteins. Of note are transglutaminase 2 (TG2), sigma 1 R (Sig-1R), pyruvate dehydrogenase kinase-4 (PDK-4) and ATPase family AAA domain-containing protein-3 (ATAD3A). TG2 interacts with GRP75 regulating MERC number, Ca^2+^ flux and protein composition [19]. Sig-1R (a ligand- operated chaperone), accumulates at MERCS and promotes the stabilisation of IP_3_R, prolonging Ca^2+^ signalling into the mitochondria [20, 21]. In skeletal muscle, further levels of regulation are achieved via PDK-4, which interacts with GRP75–IP_3_R–VDAC complex at MERCS, and is required for mitochondrial-associated membrane (MAM) formation [21]. Finally, ATAD3A, an AAA + ATPase located to the inner mitochondrial membrane (IMM) and also enriched in MERCS, can interact with OMM and ER-resident proteins, including MFN2, Drp1 and BiP via the cytosolic protein WASF3 [22, 23]. ATAD3A has roles in hormone-dependent MERCS increase and mitochondrial dynamics [23–26]. Regulatory proteins allow sophisticated control over the size, dimeter and homoeostatic functions of MERCS allowing for highly dynamic responses.

## Functions of ER-mitochondria contact sites

**Fig. 2:**
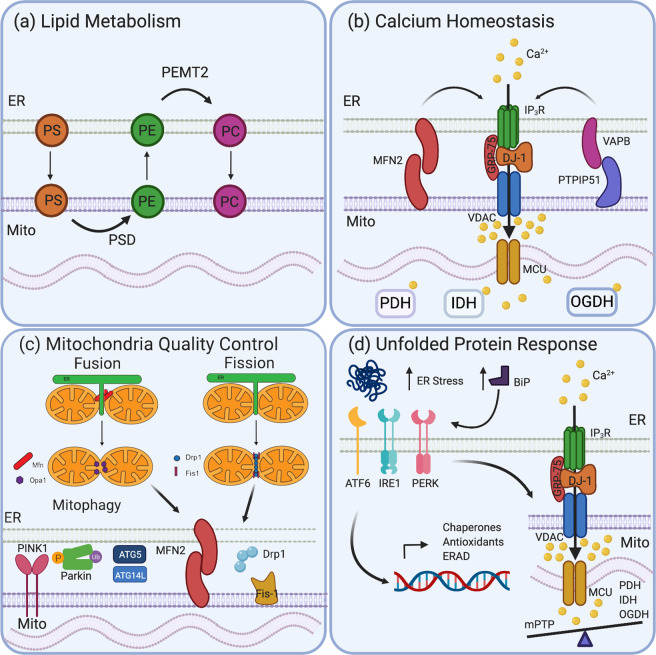
Cellular functions of mitochondria-ER contact sites. **a** Lipid metabolism requires the transfer of phospholipids from the ER to mitochondria and back again at MERCS. Phosphatidylserine (PS) in the ER is transferred to mitochondria where it is converted to phosphatidylethanolamine (PE) by enzyme PS Decarboxylase (PSD). PE is shuttled back to ER where PE-N-methyltransferase (PEMT) modifies it to phosphatidylcholine (PC). **b** Ca^2+^ homoeostasis underpins many MERCS functions and is essential to maintain cellular health. The tetramer complex IP_3_R, VDAC, GRP75 and DJ-1 allow transfer of Ca^2+^ from ER through IP_3_R and VDAC to the inter mitochondria membrane space, producing Ca^2+^ hotspots. These hotspots activate MCU, allowing Ca^2+^ into the mitochondria matrix that promotes enzymes involved in ATP production, such as PHD, IDH and OGDH. **c** MERCS impacts on MQC pathways. Both mitochondria fission and fusion require the ER and occur at MERCS. Mitochondrial fusion allows the mixing of damaged and healthy mitochondria components, diluting the damage and helping to maintain the overall health of the mitochondria. Mitochondrial fission can protect the mitochondria network by segregating highly damaged sections, promoting mitophagy. Mitochondrial fission genes *Drp1* and *Fis-1* and mitochondrial fusion genes, *MFN1/2* and *OPA1* regulate changes in mitochondrial morphology. MFN2 is found in MERCS, and it is established that the ER constricts the mitochondria, allowing oligomerisation of Drp1 around the mitochondria. MERCS is also involved in mitophagy; both PINK1 and Parkin are found in MERCS under mitophagy induction, as well as key autophagy components ATG5 and ATG14L. **d** The accumulation of unfolded proteins in the ER increases ER stress and upregulates the chaperone protein BIP that initiates the UPR. BiP activates three pathways of the UPR: ATF6, IRE3 and PERK. Activation of these receptors activates chaperone proteins, antioxidant response proteins and ER-associated protein degradation (ERAD) machinery. ER stress can increase Ca^2+^ import into the mitochondria to increase the efficiency of Ca^2+^-dependent enzymes PDH, IDH and IGDH required in ATP production, providing energy for the chaperone machinery. A balance is required, however, as high Ca^2+^ influx into mitochondria, caused by severe ER stress, can trigger mitochondria permeability transition pore (mPTP) opening, leading to mitochondrial swelling and the initiation of apoptosis.

The molecular make-up of MERCS can greatly affect its function, with specific tethers acting on specific functions, including lipid metabolism, Ca^2+^ homoeostasis, MQC as well as ER stress and the UPR. These functions are crucial in cellular survival and proliferation (Fig. 2).

### Lipid transfer

MERCS is key to several lipid metabolism pathways and is required for the transfer of lipids from the ER to the mitochondria and back again. The main lipid metabolism pathway starts when ER- produced phosphatidic acid (PA) is converted by phosphatidylserine synthase (Pss1/2) to phosphatidylserine (PS) in the ER. To form other phospholipids such as phosphatidylethanolamine (PE), PS is shuttled from the ER to the mitochondrial inner membrane space via MERCS, where it is decarboxylated by PS decarboxylase (PSD) to PE. This can then be further modified in the ER by PE-*N*-methyltransferase (PEMT) to phosphatidylcholine (PC). As PSD is only found in the mitochondria, MERCS is extremely important in the production of PE, which constitutes a large proportion of the membranes within the cell [27]. The transfer of PS from the ER to the mitochondria via MERCS is the rate-limiting step in this lipid biogenesis pathway, and thus is vital to the maintenance of the cellular and mitochondrial phospholipid balance [28].

MERCS is also involved in the metabolism of other lipids, including cholesterol. Mass spectrometry revealed that Caveolin-1, a protein involved in cholesterol distribution and organisation, is the main component of MAM fractions. Furthermore, MERCS has a higher percentage of cholesterol and sphingolipids compared with bulk ER membranes, helping to stabilise the membranes [29–31]. Thus, lipid metabolism is a key function of MERCS and important in overall cell health.

### Calcium homoeostasis

Ca^2+^ is an important second messenger in many cellular pathways. Ca^2+^ signalling relies on a low cytosolic Ca^2+^ concentration ([Ca^2+^]), which can be achieved through entry of Ca^2+^ into the mitochondria via MERCS. It has been proposed that Ca^2+^ exits the ER via IP_3_R and enters the mitochondria through VDAC, producing a Ca^2+^ hotspot [32]. This hotspot is one order of magnitude greater than cytosolic [Ca^2+^], allowing the threshold for fast Ca^2+^ transfer into the mitochondria matrix via the highly selective low-affinity Ca^2+^ channel MCU in the IMM [33, 34]. This is important as enzymes such as isocitrate dehydrogenase and oxoglutarate dehydrogenase (required in Krebs cycle) and pyruvate dehydrogenase (required in glycolysis) are situated in the mitochondria matrix and are Ca^2+^-dependent [35]. Perturbing Ca^2+^ shuttling through MERCS can decrease ATP production and the oxygen consumption rate [36, 37]. However, a fine balance of Ca^2+^ entry into the mitochondria is required, as elevated mitochondria matrix Ca^2+^ can result in increased mitochondria membrane permeability and damage to the mitochondria itself. This process is mediated by the mitochondrial permeability transition pore (mPTP), a non-specific high-conductance, voltage- dependent channel. The activation of this channel causes the mitochondrial membrane to become permeable to proteins of up to 1,500 Da, causing swelling and OMM rupture, allowing the release of pro-apoptotic factors from the mitochondria, uncoupling of oxidative phosphorylation and increased reactive oxygen species (ROS) [38, 39].

### Mitochondria quality control

There are a range of MQC mechanisms that protect against mitochondrial insult. These include the regulation of mitochondria morphology, mitochondria microtubule dynamics and mitophagy [40]. An early MQC mechanism is the regulation of mitochondria morphology via pro-fusion and pro-fission proteins. Mitochondrial fission occurs at MERCS and is facilitated by the ER enveloping the mitochondria, and thus constricting it [41]. This constriction allows the oligomerised dynamin- related protein-1 (Drp1), a pro-fission protein, to translocate to the mitochondria, enabling further constriction by Dynamin 2 and fission of the mitochondrial network [41]. Furthermore, both MFN2 and MFN1 are pro-fusion proteins and key MERCS tethering proteins, suggesting a strong link between the two [8–11]. It has recently been reported that fusion machinery, including MFN2, converges at MERCS aiding mitochondria fusion at these sites [42]. These data demonstrate that MERCS is a key location for both fission and fusion of the mitochondria network and is intrinsically linked to MQC.

Mitophagy, the bulk degradation of mitochondria, is also linked to MERCS. Pre-autophagosome markers ATG14L and ATG5, key autophagy components, are localised to MERCS upon starvation- induced mitophagy [43]. Studies in yeast also found that efficient mitophagy depends on MERCS tethers, and that the yeast tether, ERMES, co-localises with the sites of autophagosome biogenesis [44]. In accordance with this, the flux of Ca^2+^ from the ER to the mitochondria through MERCS is required for both starvation and PINK1/Parkin-induced mitophagy [45]. Further studies have revealed that the mitophagy machinery uncouples the mitochondria from the ER via destruction of MFN2, which is an early target of PINK1/Parkin-mediated phosphoubiquitination, facilitating mitophagy [46]. This demonstrates that uncoupling and coupling of ER and mitochondria membranes are both required for efficient mitophagy, and that MERCS has a key role in maintaining efficient MQC mechanisms and mitochondrial health.

### ER stress and unfolded protein response (UPR)

MERCS has also been connected with ER stress and UPR, an intracellular signalling pathway that is activated by the accumulation of unfolded proteins in the ER [47]. Under basal conditions, ER- residing chaperone proteins, including BiP, hold proteins in the ER, allowing them to fold correctly. An accumulation of unfolded proteins can result in ER stress that is detected by BiP and initiates the UPR [48]. The UPR is predominantly composed of 3 major pathways: PERK, IRE1 and ATF6. Once activated, they allow the upregulation of chaperone proteins and prevent further translation of proteins. ER stress has been shown to increase Ca^2v^ uptake into the mitochondria, increasing ATP production and providing energy for the chaperone machinery to aid in the folding of proteins, thereby preventing ER stress and apoptosis [49]. These initial responses to ER stress have been reported to be accompanied by an increase in MERCS [50]; however, when the ER stress becomes too severe, apoptosis can also be induced through mPTP and MERCS [51]. Interestingly, two downstream pathways of the UPR have been associated with MERCS. Firstly, PERK has been shown to directly interact with MFN2, this interaction being required for the progression into the UPR [52], with PERK silencing indicating weakened MERCS and protection against ROS damage [53]. More recently, IRE1α was discovered as a novel substrate for MITOL, an OMM E3 ubiquitin ligase that can regulate MERCS [54]. MITOL can block IRE1α-mediated mRNA decay and MITOL loss enhances IRE1α-dependent apoptosis in a MERCS-dependent manner [55]. These studies suggest that MERCS and the UPR are closely linked, and that MERCS can regulate the health of ER, however, their exact role is still under investigation.

## ER-mitochondria contact sites in neurodegeneration

### Alzheimer’s disease

**Fig. 3:**
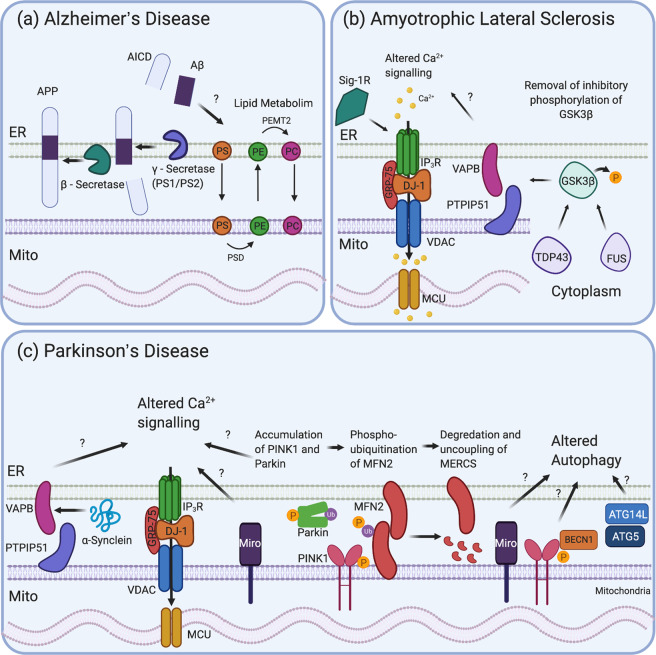
Mitochondrial ER contact sites in neurodegeneration. **a** AD: amyloid precursor protein (APP), along with its metabolites, and β- and γ-secretase enzymes are found in MERCS. The APP is first cleaved by β-secretase and then γ-secretase to release Aβ. Aβ has been shown to alter lipid metabolism at MERCS. **b** ALS: TDP-43 and FUS in the cytoplasm activate GSK3β by dephosphorylating it at cysteine 9. GSK3β can then disrupt VAPB and PTPIP51 binding, uncoupling the ER from the mitochondria and altering Ca^2+^ signalling. Sig-1R can bind IP_3_R, stabilising it in the membrane, while loss of Sig-1R can result in ALS-like symptoms in mice and uncoupling of MERCS. **c** PD: WT or mutated α-Synuclein interacts with VAPB altering its binding to PTPIP51, disrupting MERCS and Ca^2+^ signalling. Miro is present in MERCS and can disrupt Ca^2+^ signalling and autophagy. PINK1 and Parkin, under mitophagy induction, have been shown to localise to MERCS and also impact on Ca^2+^ signalling and to promote the phosphoubiquitination of MFN2, resulting in its degradation and the uncoupling of the mitochondria from the ER. PINK1 and Miro have also been associated with altered mitophagy as PINK1 interacts with BECN1. Key autophagy genes (e.g., *ATG14L and ATG5*) are also present in MERCS and impact on mitophagy, a pathway dysregulated in PD.

AD studies were among the first to demonstrate a link between neurodegeneration and MERCS. The key pathologic hallmarks of AD are aberrant protein aggregates of hyperphosphorylated Tau and extracellular plaques of β-amyloid (Aβ). Aβ40 and Aβ42 are the main component of extracellular plaques and are the result of abnormal cleavage of amyloid precursor protein (APP) by Presenilin 1 (PS1) and Presenilin 2 (PS2), active components of the γ-secretase complex. *APP* and *PS1/2* are the principal genes associated with familial AD [56]. PS1 and PS2 are enriched at MAMs correlating with an increase in γ-secretase activity [57]. In addition, APP and β-secretase are also localised and processed at MERCS [58, 59]. Mutations in *PS1, PS2* or *APP* result in increased MERCS proximity and enhanced lipid metabolism [60, 61]. In vitro and in vivo models of AD have also been investigated for alterations in MERCS, but the impact in AD is still disputed. Exposure of oligomeric Aβ in primary hippocampal neurones increases MERCS and alters Ca^2v^ homoeostasis [62], while familial or sporadic AD patient fibroblasts have increased ER-mitochondria coupling and MERCS function, including phospholipid and cholesterol metabolism [61]. However, dynamic and ultrastructural analysis of hippocampal neurones from AD rat models showed a decrease in MERCS, correlating with a reduction in lipid metabolism and specific alterations in mitochondria lipids [63]. In *Drosophila*, forced expression of an artificial linker, that increases MERCS in vivo, rescues locomotion and prolongs the survival of AD models [64]. Studies of AD mice have shown changes in the proteasome occurring in MERCS in the early stages of amyloid accumulation that predate symptoms of AD, such as memory loss [65]. This leads to the hypothesis that MERCS dysregulation may impact the progression of AD. This is plausible as many functions of MERCS are prerequisites for neurodegeneration. Overall, however, many of these results remain contradictory (Fig. 3a).

### Parkinson’s disease

There is growing evidence that MERCS also plays an important role in PD pathogenesis. Firstly, α-Synuclein, the most common component of Lewy bodies, a hallmark of PD, can localise to MERCS, with WT α-Synuclein associating more with MERCS than PD-related mutations [66]; however, how α-Synuclein influences MERCS is disputed. α-Synuclein has been reported to interact with VAPB, with overexpression of α-Synuclein WT, A30P or A53T causing a decrease in VAPB and PTPIP51 binding correlating with a reduction in MERCS, Ca^2+^ flux and ATP production [67]. Others have reported that α-Synuclein and its pathogenic mutations enhance MERCS and Ca^2+^ transfer to mitochondria in HeLa cells [68, 69]. This lack of consensus is likely due to the method of analysis and cell types used. There is, however, little doubt that α-Synuclein is present at MERCS and can lead to dysfunction of the ER or mitochondria in PD.

Secondly, familial linked PD genes have also been associated with MERCS. As described previously, the PD-related protein DJ-1 has been shown to interact with VDAC, GRP75 and IP_3_R in a tetramer complex, and is thought to facilitate Ca^2+^ homoeostasis, as well as acting as a functional tether. Ablation of DJ-1 decreases MERCS association and alters Ca^2+^ handling, which can be rescued with WT but not PD-related mutant DJ-1 [5, 6]. Furthermore, both *PINK1* and *Parkin*, two genes mutated in autosomal recessive PD, acting together to regulate MQC mechanisms, have been found to localise to MERCS under mitophagy induction [70]. Induced pluripotent stem cell-derived dopaminergic neurones show Parkin-dependent uncoupling of the ER and mitochondria [46]. PINK1 interacts with BECN1, a key component of the class III phosphatidylinositol 3-kinase complex required for omegosome generation, and localises at MERCS [70]. Overexpression of Parkin was shown to significantly increase MERCS in Hela cells, which correlates with enhanced Ca^2+^ signalling and ATP production [71]. However, reports have been conflicting, as *PARK2-* knockout mice and patient cells showed an increase in the proximity of the ER and mitochondria and altered Ca^2+^ flux [72], a result that was supported in *Drosophila* models [73]. More recently, however, loss of Parkin in human fibroblasts resulted in a decrease in MERCS [74]. These data demonstrate a clear role for PINK1 and Parkin in MERCS, but the exact details are yet to be understood.

Many PINK1- and Parkin-associated proteins have also been implicated in MERCS, including MFN2 and Miro. MFN2 (a key tether in MERCS) can be phosphoubiquitinated in a PINK1-/Parkin- dependent manner under induction of mitophagy, and degraded, resulting in an uncoupling of the ER from the mitochondria [46]. This was supported by studies in *Drosophila* and human fibroblasts, where loss of Parkin or ubiquitination-preventing mutations in MFN2, reduced MERCS, implying that PINK1/Parkin can regulate MERCS through MFN2 [74]. Miro, a target for PINK1/Parkin in mitochondrial transport along microtubules, has also been linked with MERCS [75, 76]. Previously, Gem1 (the yeast homologue of Miro) was shown to be a regulator of the ERMES complex, a known MERCS tether in yeast [77]. However, Miro has also been reported to have functions outside of mitochondrial dynamics, including regulating Ca^2+^ transport through MERCS [78]. A more direct link has recently been established as two studies identified novel mutations in *RHOT1*, the gene that encodes Miro, in PD patients. All four novel *RHOT1* mutations showed attenuated Ca^2+^ handling, autophagy and a decrease in MERCS in patient-derived fibroblasts [79, 80]. Thus, Miro can influence MERCS, and may lead to dysfunctional ER or mitochondria in PD (Fig. 3c).

### Amyotrophic lateral sclerosis

ALS is a fatal neurodegenerative disease. Familial forms of ALS account for 10% of all cases with mutations in *SOD1*, *C9ORF72*, *FUS* and *TARDBP* occurring most frequently [81]. Many of these ALS-related genes and others, such as *VAPB*, have been associated with altered signalling at MERCS. Specifically, mutations in VAPB cause an autosomal-dominant form of ALS and increase the affinity to its binding partner PTPIP51. This was shown to correlate with increased Ca^2+^ transfer into the mitochondria [12]. Furthermore, it has been demonstrated that both WT TDP-43, ALS- related TDP-43 and FUS expression perturbs MERCS, which can disturb VAPB–PTPIP51 binding through glycogen synthase kinase 3β (GSK3β) regulation [13, 14], causing impairment of Ca^2+^ uptake into the mitochondria and ATP production [13, 14]. Other studies have linked Sig-1R (an ER protein that facilitates Ca^2+^ transfer by binding IP_3_R in MERCS) to familial ALS [82] as loss of Sig-1R can uncouple ER from the mitochondria [83]. Mouse models have supported this with Sig-1R null mice displaying features of ALS, while treatment with Sig-1R agonists was beneficial in some ALS models [83, 84]. Furthermore, disruption of Sig-1R in SOD1 G85R mice accelerated disease onset while also disrupting MERCS integrity [85]. These data suggest a link between MERCS and ALS (Fig. 3b).

## Functional genetic screens

Functional genomics link gene sequence to function and have been key in understanding molecular mechanisms of disease. Combined with genetic screening approaches, functional genomics is a powerful tool. The introduction of RNAi and CRISPR/Cas9 has revolutionised genetic screening due to ease of use, high efficiency, reliability and cost-effectiveness [86, 87]. Here we will discuss how these tools may be used to elucidate the role of MERCS in neurodegenerative diseases.

### RNAI vs CRISPR/Cas9

**Fig. 4:**
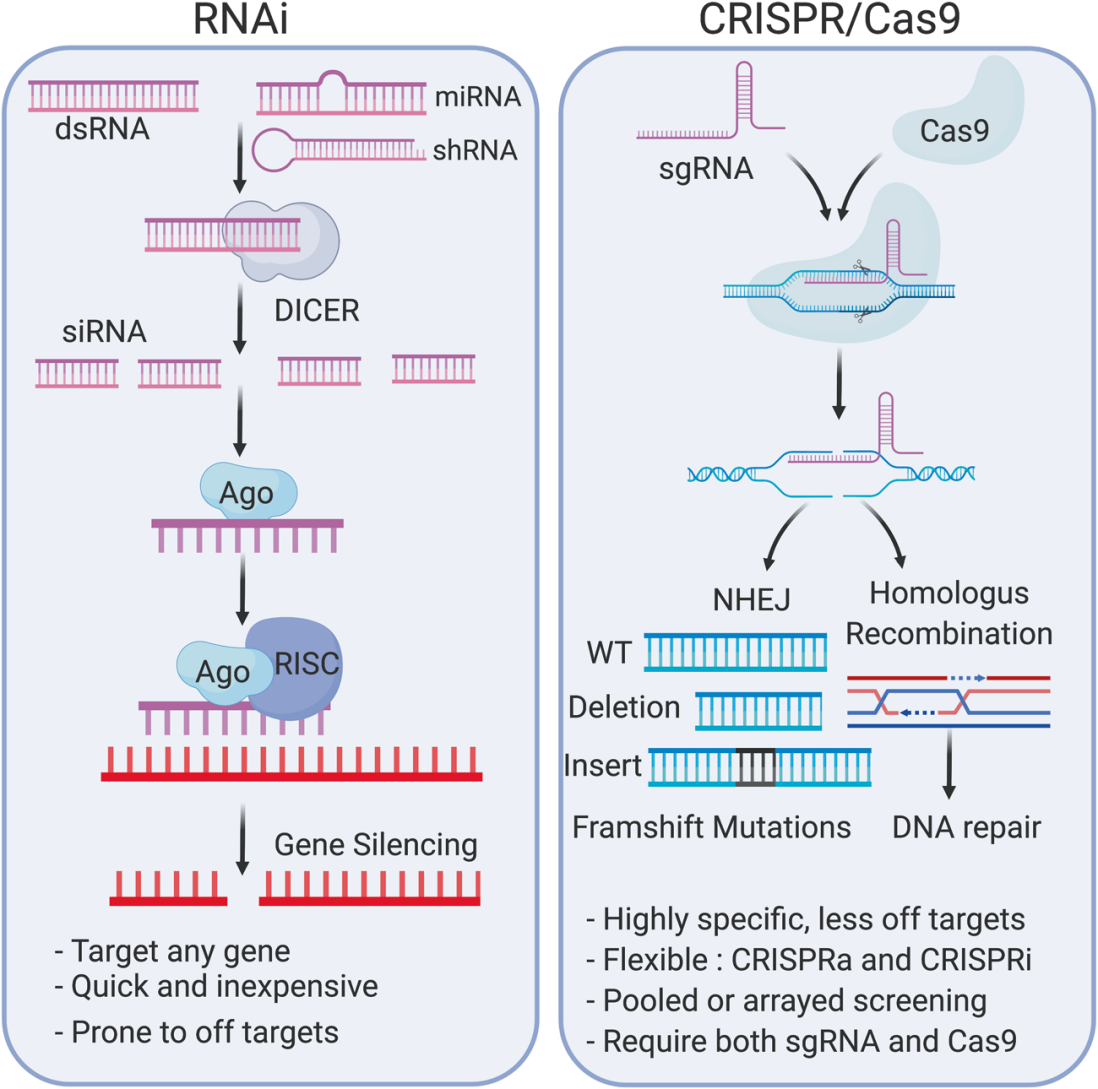
Mechanisms for RNAi and CRISPR/Cas9 gene silencing techniques. In RNAi knockdown, the presence of double-stranded RNA (dsRNA), microRNA (miRNA) and short-hairpin RNA (shRNA) in the cell initiates recruitment of the ribonuclease DICER. DICER cuts dsRNA into shorter fragments called small-interfering RNA (siRNA). Argonaut is a key component of the RNA-induced silencing complex (RISC) that can bind non-coding RNAs, including siRNAs, and recruit the remaining RISC complex components. The RISC complex identifies mRNA of interest, via complimentary base pairing, and cleaves it, inhibiting translation of that protein resulting in a knockdown. The CRISPR/Cas9 system utilises the adaptive immune response of bacteria to conduct genome editing. Both Cas9 endonuclease, as a vector or protein, and gRNAs are required for this process. gRNAs (aka sgRNAs) are short synthetic RNA sequences composed of a tracrRNA, a scaffold sequence necessary to bind Cas9, and crisprRNA (crRNA) (a user-defined 20-bp nucleotide sequence complementary to the gene of interest (GOI)). Through complementary base pairing of the crRNA, the Cas9 is directed to specific genomic locations where it creates double-stranded breaks (DSB). Two principal mechanisms repair DSB: non-homologous end joining (NHEJ) and homologous recombination (HR). NHEJ is error prone and can result in insertions and/or deletions (INDELS) in the genome, causing frameshift mutations and leading to premature stop codons and gene knockout. The HR pathway uses template DNA and DNA synthesis machinery to repair the DNA without error, and can be utilised to incorporate point mutations or other genes (knock-ins).

For the last decade, RNAi has been the principal approach for functional genomic screening. RNAi is a conserved endogenous pathway that uses the ribonuclease DICER to cut double-stranded RNA into small-interfering RNA (siRNA). The siRNA utilises base complementation in conjunction with RNA-induced silencing complex (RISC) to degrade mRNA, reducing protein levels [88]. Artificially, siRNA can be designed to target any mRNA sequence for any gene of interest (GOI), making it quick to develop a range of RNAi libraries (Fig. 4) [82]. More recently CRISPR (clustered regularly interspaced short palindromic repeat) in combination with Cas9, an RNA-guided endonuclease [89, 90], has been used to conduct highly specific genome editing in mammalian cells [91, 92]. CRIPSR/Cas9 uses guide RNA (gRNA) to target Cas9 to a specific region of the genome. Once Cas9 induces double-strand breaks in DNA backbone, this can be repaired by two main DNA repair pathways: non-homologous end joining (NHEJ) or homologous recombination. NHEJ is error prone and results in insertion and/or deletion (INDELS) in the DNA, shifting the reading frame, to produce premature stop codons [87]. The major difference between RNAi and CRISPR/Cas9 is that RNAi produces knockdown (KD) of the GOI, while CRISPR/Cas9 results in knockout (KO). While incomplete KD may cause experimental issues, complete KO may be detrimental to cell survival [93]. Furthermore, siRNAs have reduced base complementarity and are prone to more off- target effects than the CRIPSR/Cas9 system [94–96]. CRISPR/Cas9 does have its disadvantages requiring both gRNA and Cas9 to conduct editing, making it more time consuming than siRNA, and the gRNAs cannot be targeted anywhere as they have to include a protospacer-adjacent motif (PAM) site. However, these are numerous within a genome and this is rarely a limiting factor. Overall, the benefits of CRISPR/Cas9 outweigh the disadvantages, and now CRISPR/Cas9 systems are used routinely in functional genomic studies and HTS.

### Screening strategies

**Fig. 5:**
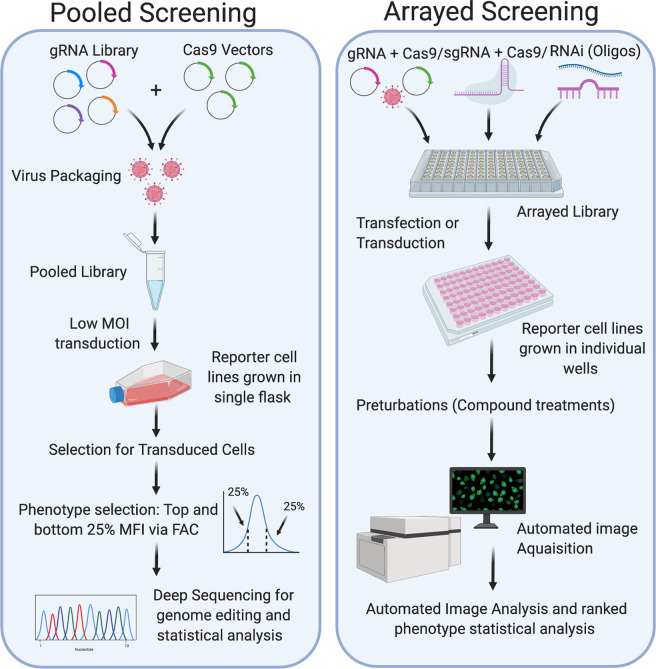
Workflow for pooled and arrayed screening approaches. Pooled screening requires the gRNA library and Cas9 to be delivered into cells within a single vessel (using a low MOI viral transduction to maintain a 1:1 ratio of gRNA to cells). The cells then undergo selection for transduced cells and further selection for the phenotype of interest. For example, mean fluorescence intensity (MFI) where the top and bottom 25% are examined for survival. The output for a pooled screen comes from deep sequencing of the genomic DNA from control vs treated samples. Statistical analysis of gRNAs that are enriched or depleted is conducted. Arrayed screens can be conducted with a wider range of silencing techniques, including CRISPR/Cas9 or RNAi. This is because the final readout comes from phenotypic analysis, rather than deep sequencing of genomic DNA. Individual gRNAs and siRNAs are delivered (by transduction or transfection) to cells in specific wells of a 96-well plate (eliminating the possibility that more than one gRNA or si9RNA could be delivered per cell). The transfected cells remain in an arrayed format for phenotype analysis, and statistical analysis in which phenotypes of interest can be matched with gRNA/siRNA by their position in the plate.

Two principal screening strategies are used: arrayed and pooled (Fig. 5). In an arrayed format, reagents are arranged in multiwell plates with cell targeting of gRNAs or siRNAs occurring in individual wells. Each step of an arrayed screen requires a multiwell approach, meaning it can be expensive and labour intensive, often requiring specialised automated equipment [86, 87]. The readout from an arrayed screen can vary and is often related to a phenotype of interest, with common readouts involving fluorescence [97] and high-content image analysis [98]. Pooled screens, however, combine reagents in one complex mixture and are conducted on a single pool of cells. To prevent multiple gRNAs entering a cell, a low ratio of virus to host cells is used (termed “multiplicity of infection” (MOI)). Further selection is often required with pooled screens to select for a phenotype of interest, for example, treatment with a specific drug or disease mutation that can be compared to an unselected control pool. The readout for pooled screens is DNA sequencing, which reveals the enriched or depleted gRNAs in the cell population. Confirmation can be achieved when multiple gRNAs for the same gene are enriched. Pooled screens are quicker, cheaper and less labour intensive than arrayed screens. However, which screening strategy to use depends on a range of factors, including the phenotype of interest and the readouts available, the size of the screen (whole genome vs druggable/subset) and the cost and efficiency of targeting.

## Studying mitochondria-ER contact sites

Owing to its small size and high complexity, studying MERCS is difficult, but recent technological advances can be coupled with HTS and offer new opportunities for investigation.

### Electron microscopy (EM) and super-resolution microscopy techniques

EM is the gold standard for visualising MERCS as it can resolve the close apposition of the ER and mitochondria membranes (10–30 nm); however, EM requires that samples must be fixed and processed, making it difficult to collect multiple images and thus inappropriate for HTS. Super-resolution techniques have also been used to visualise MERCS, including stimulated emission depletion (STED), photo-activation localisation microscopy (PALM), stochastic optical reconstruction microscopy (STORM) and structural illumination microscopy (SIM) [99–103]. However, super-resolution techniques are also inappropriate for HTS. PALM and STORM collate many images and lead to low temporal resolution, while SIM and STED require specialised, expensive equipment that is not yet automated. These techniques also require a range of fixation or mounting processes, making them difficult to conduct on a large number of samples.

### Proximity ligation assay

**Fig. 6:**
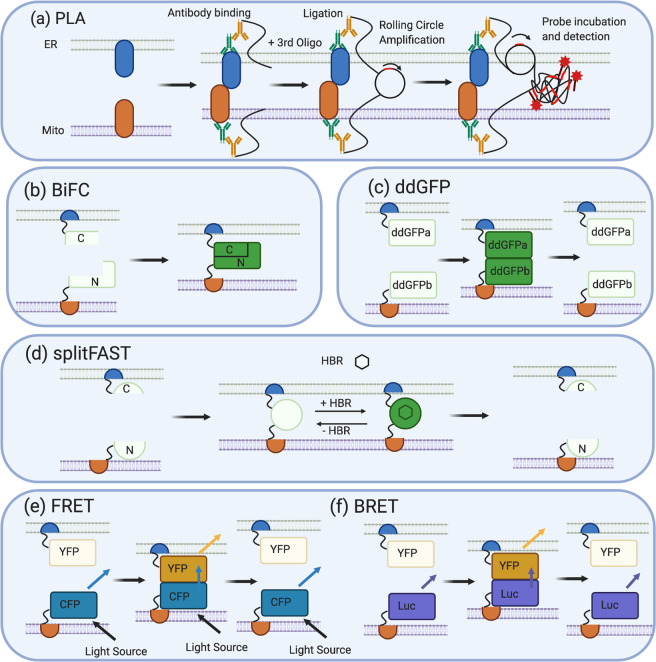
Fluorescence-based techniques to visualise MERCS. **a** PLA: the sample is fixed and incubated with primary antibodies targeting proteins in MERCS (usually MERCS tethers) and a modified secondary antibody (which is attached to an oligonucleotide strand). If the two proteins are in close proximity, as they are in MERCS, the addition of a third oligonucleotide results in complementary base pairing between the three oligonucleotides and the formation of circular DNA. The circular DNA can undergo rolling circle amplification, creating a long strand of DNA and multiple copies of the circular DNA. Fluorescent probes are designed to be complementary to a sequencing within the circular DNA. The probes hybridise in multiple regions, allowing the visualisation of the fluorescent signal and MERCS. **b** BiFC also utilises proximity to visualise MERCS. Two non-fluorescence fragments of a fluorescence protein, commonly GFP or Venus, are fused to transmembrane domains, or whole proteins, found in the ER or mitochondrial membrane. If MERCS does not form the BiFc, constructs remain separate and do not fluoresce, but when MERCS forms, the ER and mitochondria membrane come into close proximity bringing with them the two non-fluorescent fragments, allowing them refold into whole GFP or Venus protein whose fluorescence can be visualised and measured. **c** Similarly, the ddGFP system is fused to protein fragments or whole proteins found in ER and mitochondria membrane; however, each protein is fused to non-fluorescence ddGFP monomers. One monomer contains a chromophore that is destabilised and quenched, while the other completely lacks a chromophore. When MERCS forms, these ddGFP monomers come into close proximity, heterodimerise and complement each other, allowing fluorescent detection. As there is no protein folding, this process is reversible. **d** splitFAST utilises the 14- kda protein (FAST), which is split into N- and C-terminal fragments. These fragments can be attached to two interacting proteins or membrane fragments of a protein. For the study of MERCS, these interacting proteins would be in the ER or mitochondrial membrane. When MERCS forms, the N and C fragments combine, and upon the addition of HBR, they fluoresce allowing visualisation of MERCS. This is reversible, as HBR can be added or removed, but the FAST protein can also dissociate when the ER and mitochondria membrane are not in close proximity. **e** FRET: a FRET donor (CFP) and FRET acceptor (YFP) are fused to resident ER or mitochondrial proteins or transmembrane protein fragments. When ER or mitochondrial membranes are not in close proximity, a light source illuminates the CFP donor but the YFP acceptor is not close enough for FRET to occur and so blue light emitted. When MERCS forms, the FRET donor (CFP) and acceptor (YFP) are in close proximity, so FRET can occur, and blue light is transferred to YFP and yellow fluorescence is emitted. This is reversible as no protein folding or contact occurs between FRET pairs, **f** BRET also employs FRET to visualise MERCS; however, rather than CFP, the resident ER, mitochondria protein or transmembrane fragments are fused to a luciferase enzyme that acts as a light source. When MERCS forms, the luciferase enzyme and FRET acceptor are in close proximity, and so energy transfer can occur from the luciferase enzyme and YFP, which is emitted as yellow florescence. This is reversible as no protein folding occurs.

A highly sensitive approach, termed in situ Proximity Ligation Assay (PLA), has been used to investigate endogenous protein interactions [104] and optimised to study MERCS [105]. This is a probe-based system where the endogenous proteins of interest are targeted by primary antibodies, then by specialised secondary antibodies fused to oligonucleotides. If the two endogenous proteins are in close proximity, then, on the addition of a third oligonucleotide, there is complementary base paring and the formation of circular DNA. The circular DNA can undergo rolling DNA replication creating many repeats. This can be visualised using complementary fluorophore-labelled probes (Fig. 6a) [106]. This technique is highly specific as it uses duel recognition from primary and secondary antibodies, it can be conducted on multiple conditions as the readout is fluorescence and specialised imaging equipment is not needed [105]. However, false detection rates are high; therefore, isotope antibody controls are routinely used to detect background fluorescence from potentially unspecific primary antibodies [107, 108]. In PLA, the detection of fluorescence without the need for fluorescent proteins decreases the potential for artefacts, meaning readouts are easily measured in either pooled or arrayed screens. However, the sample preparation requires fixation and multiple incubation steps, making it highly costly and labour intensive. Furthermore, the range of detection for PLA is between 40 and 60 nm, greater than the size of most MERCS, hence, it is showing protein proximity rather than protein–protein interactions; however, this is greater than the resolution gained from a traditional fluorescence microscope and simple co-localisation analysis [109]. Thus, while the practicality of PLA in HTS is limited, it could be suitable for hit validation.

### Split fluorescence reporters of contacts

Like PLA, other methods that rely on construct proximity are split FP systems, including biomolecular fluorescence complementation (BiFC), double-dimerising GFP (ddGFP), fluorescence resonance emission transfer (FRET) and bioluminescence resonance emission transfer (BRET).

#### Biomolecular fluorescence complementation (BiFC)

Typically, in BiFC, a FP is split into two non-fluorescent fragments that are fused to the proteins of interest. With regard to MERCS, they are tethered to either resident ER or mitochondrial proteins. If the two membranes come into close proximity, then the two complementary fragments of the FP will reassemble, restoring the fluorescence of the chromophore [110]. A range of BiFC constructs using different FP have been developed, including split mVenus, spGFP1–10/spGFP11 and dsRFP [111, 112]. These have been successfully used to investigate novel proteins associated with MERCS [113–115]. The simplicity of the fluorescence readout makes BiFC ideal for HTS and has been used previously in large-scale protein–protein interaction studies [116–118]. A major disadvantage of BiFC technique is that it requires folding of two FP fragments that are thermodynamically stable and adverse to unfolding [119]. This may limit screening investigating increases in MERCS. Furthermore, BiFC systems are prone to spontaneous assembly that may alter basal stress levels in the cell (Fig. 6b) [120].

#### Double-dimerising green fluorescent protein (ddGFP)

The ddGFP system has also been used to investigate MERCS and involves the reversible binding of two non-fluorescent, “dark”, ddGFP monomers, ddGFPa and ddGFPb, to produce a fluorescent heterodimer. ddGFPa is mutated (I11L and S14A and K163G) such that the chromophore is destabilised and quenched, while ddGFPb completely lacks a chromophore. When ddGFPa heterodimerises with ddGFPb, complementation occurs and a fluorescent heterodimeric complex is formed [121, 122]. This system, like BiFC, can be fused to a variety of proteins of interest and has been used to study protein–protein interactions (Fig. 6c) [11, 121]. The advantages of this method are similar to BiFC, namely ease of use, a simple readout and low labour intensity with the added advantage of requiring no protein folding in order to obtain the fluorescent readout. However, ddGFP systems tend to have intrinsically low fluorescence that may limit the utility of these constructs in screening as it would limit the assay range. One solution would be the use of an adapted split FP system such as splitFAST. SplitFAST relies on the binding of a 14-kDa protein Fluorescence-activating and Absorption Shifting Tag (FAST) to a HydroxyBenzylidene Rhodanine (HBR) analogue. HBR analogues are weakly fluorescent in solution, but the binding of FAST precipitates them and increases their fluorescence in a reversible manner [123]. SplitFAST alleviates the problem of reversibility and spontaneous assembly of BiFC system and low fluorescence output of ddGFP system while maintaining the easy readout and low labour intensity of split FP systems, which make them ideal for HTS of either type (Fig. 6d).

#### FRET- and BRET-based systems

FRET is a collision-free, but distance-dependent, process that involves the transfer of energy from one excited donor fluorophore to a suitable acceptor fluorophore [124]. FRET fluorophore pairs have been fused to resident ER and mitochondrial proteins and used as an interaction-free way to detect MERCS [11, 74]. A major advantage over BiFC is there is no requirement for protein folding and so it is thought to be reversible. Furthermore, FRET is intrinsically sensitive to molecular distance, as energy transfer is proportional to the distance between the two fluorophores, thus giving a fluorescence readout for the distance between the two membranes (Fig. 6e) [11, 74, 124]. Rapamycin-inducible FRET-based probes are an example of a successful FRET tool used to visualise MERCS [125]. The addition of rapamycin allows maximal energy transfer between FRET pairs, enabling quantitative measurements of contact distance; however, equimolar concentrations of the FRET pairs are required as FRET is affected by the relative amount of each florophore [125]. This was skilfully overcome by transfection of one plasmid containing both acceptor and donor constructs separated by TAV2a sequence (which self-cleaves), achieving equal expression of both FRET pairs, and has been used in conjunction with rapamycin to investigate potential MERCS proteins, including MFN2 and Parkin [11, 74]. In theory, FRET probes would be ideal for HTS; however, in practice, FRET-based assays have a low signal-to-noise ratio, limiting reliability of the FRET values and, due to the need for an external light source, are prone to photobleaching. BRET is a variant of FRET, which does not require an external light source, as the donor fluorophore consists of a luciferase enzyme. The luciferase enzyme oxidises the substrate bioluminophore providing light for energy transfer to the acceptor [126]. Internal illumination has two main benefits: firstly, it reduces phototoxicity and bleaching, thereby decreasing noise previously seen with FRET. Secondly, the orientation of acceptor and donor fluorophores is less essential, making it easier for energy transfer to occur (Fig. 6f). Together, this makes BRET robust and more sensitive, which is beneficial in either pooled or arrayed HTS; however, only one such probe, Mitochondria-ER Length Indicator Nanosensor (MERLIN) developed by the Garcia-Saez lab, is currently fit for purpose [127].

#### Calcium-based sensors

Functional readouts, such as Ca^2+^ flux, could allow for different avenues of MERCS investigation. Many tools can be used to visualise intracellular Ca^2+^, including bioluminescent proteins such as aequorin, which upon binding of Ca^2v^, undergo a conformational change, oxidation of coelenterazine and emission of a photon at 470 nm, chemical Ca^2+^ indicators and dyes that change their emitted fluorescence when Ca^2+^ is bound or genetically encoded Ca^2+^ indicators (GECI), which contain Ca^2+^ binding protein Calmodulin, which, with the binding of Ca^2+^, changes the fluorophore environment resulting in fluorescence^128^. Previously, the combination of cytosolic (Fluro4) and mitochondria (Rhod2) fluorescent dyes has been used to investigate Ca^2+^ flux and functionality of MERCS in vitro [12–14, 128]. Single-fluorophore GECI, GCaMP, targeted to mitochondria or ER, has also been used successfully in vitro and in vivo [103, 129]. These methods are simple, have a range of fluorophores and have a fluorescence readout; however, chemical Ca^2+^ indicators require dye loading, increasing workload. Aequorin, however, has a high signal-to-noise ratio, a wide dynamic range and does not require an external illumination avoiding phototoxicity, but the recharge process, via coelentrazine, is slow and thus makes it suboptimal for HTS [130]. GECIs have previously been used with flow cytometry in a high-throughput manner and can be genetically targeted to various membranes within the cells [128, 130]. Furthermore, previous shortcomings, such as decreased sensitivity, small dynamic range compared to dyes [131] and cytotoxic accumulation of GECIs in the nucleus [132] were rectified in this study [132]. Thus, GECIs have the potential to be used as a tool in HTS of MERCS, more specifically in pooled CRISPR screening due to their ease of use with flow cytometry [133, 134].

## Conclusions

Overall, MERCS has been implicated in a range of neurodegenerative diseases, but many questions still remain unanswered. A range of different tools have been developed to study MERCS in a range of circumstances. The microscopy-based techniques, such as EM, SEM, SIM and STORM, are remarkable in their ability to visualise MERCS on the nm scale, but require specialised equipment and can only be conducted on a small number of samples; therefore, they are not suitable for screening. Both PLA and split FP use fluorescence as a readout and the proximity of membrane-bound ER and mitochondrial proteins to visualise MERCS. A downside of PLA is that it requires fixation and has multiple incubation steps, meaning it cannot be used as HTS but would likely be useful in the validation of hits. Unlike PLA, split FP can easily be conducted on a large scale as it has limited sample or data processing, specialised equipment is not needed and stable cell lines can be engineered, which dramatically reduces cost and labour. The range of split FP comes with its own advantages and disadvantages: BiFC has the simplest readout, clearest signal and least noise-associated analysis, unlike ddGFP, FRET or BRET. The advantage of FRET/BRET probes is that they do not self-assemble and are reversible, as they do not require protein folding. BRET also has reduced phototoxicity and bleaching, thereby decreasing noise previously seen with FRET. GECIs may also be suitable for investigating MERCs, these sensors do not rely on proximity of either the mitochondria or ER, but the influx of Ca^2+^ into the mitochondria from the ER. However, MERCS has a variety of functions, not only Ca^2+^ homoeostasis, and this approach may exclude potential regulators of other functional subtypes of MERCS^2^.

Overall, BRET probes would make a good choice for HTS of MERCS, but screening using the other FP systems (specifically splitFAST) would also allow for rapid discovery of novel proteins involved with MERCS regulation in neurodegenerative disease with relative ease. Furthermore, GECIs have been shown to be successful in high-throughput screening to investigating Ca^2+ +^ flux and so are also suitable for investigating MERCS. The combination of both proximity-based and functional (Ca^2+^ indicators) screening approaches would be highly beneficial in understanding both the physical and functional role of MERCS in neurodegenerative diseases.
